# Clinical mentorship to improve pediatric quality of care at the health centers in rural Rwanda: a qualitative study of perceptions and acceptability of health care workers

**DOI:** 10.1186/1472-6963-14-275

**Published:** 2014-06-20

**Authors:** Anatole Manzi, Hema Magge, Bethany L Hedt-Gauthier, Annie P Michaelis, Felix R Cyamatare, Laetitia Nyirazinyoye, Lisa R Hirschhorn, Joseph Ntaganira

**Affiliations:** 1 University of Rwanda, College of Medicine and Health Sciences, School of Public Health, Kigali, Rwanda; 2 Partners In Health, Kigali, Rwanda and Boston, USA; 3 Division of General Pediatrics, Children’s Hospital Boston, Boston, USA; 4 Division of Global Health Equity, Brigham and Women’s Hospital, Boston, USA; 5 Department of Global Health and Social Medicine, Harvard Medical School, Boston, USA

**Keywords:** Clinical mentorship, Quality improvement, Pediatrics, Health centers, Perceptions, Acceptability, IMCI, Rwanda

## Abstract

**Background:**

Despite evidence supporting Integrated Management of Childhood Illness (IMCI) as a strategy to improve pediatric care in countries with high child mortality, its implementation faces challenges related to lack of or poor post-didactic training supervision and gaps in necessary supporting systems. These constraints lead to health care workers’ inability to consistently translate IMCI knowledge and skills into practice. A program providing mentoring and enhanced supervision at health centers (MESH), focusing on clinical and systems improvement was implemented in rural Rwanda as a strategy to address these issues, with the ultimate goal of improving the quality of pediatric care at rural health centers. We explored perceptions of MESH from the perspective of IMCI clinical mentors, mentees, and district clinical leadership.

**Methods:**

We conducted focus group discussions with 40 health care workers from 21 MESH-supported health centers. Two FGDs in each district were carried out, including one for nurses and one for director of health centers. District medical directors and clinical mentors had individual in-depth interviews. We performed a hermeneutic analysis using Atlas.ti v5.2.

**Results:**

Study participants highlighted program components in five key areas that contributed to acceptability and impact, including: 1) Interactive, collaborative capacity-building, 2) active listening and relationships, 3) supporting not policing, 4) systems improvement, and 5) real-time feedback. Staff turn-over, stock-outs, and other facility/systems gaps were identified as barriers to MESH and IMCI implementation.

**Conclusion:**

Health care workers reported high acceptance and positive perceptions of the MESH model as an effective strategy to build their capacity, bridge the gap between knowledge and practice in pediatric care, and address facility and systems issues. This approach also improved relationships between the district supervisory team and health center-based care providers. Despite some challenges, many perceived a strong benefit on clinical performance and outcomes. This study can inform program implementers and policy makers of key components needed for developing similar health facility-based mentorship interventions and potential barriers and resistance which can be proactively addressed to ensure success.

## Background

There are critical challenges to the quality of care provided in sub-Saharan Africa [1,2]. Delivering high quality care is often limited by both facility challenges and the performance of health providers [3]. Several studies show that lack of knowledge about basic diagnosis and management of common diseases are often associated with ineffective or harmful health care practices [4]. Furthermore, health care workers’ report that training programs and setting goals play key roles in improving the quality of care provided [3,5].

The World Health Organization (WHO) designed the Integrated Management of Childhood Illness (IMCI) protocol to improve under-five care at first-line health centers (HCs). IMCI is an algorithmic approach to pediatric visits intended for use in countries with infant mortality of 40 per 1000 live births or greater. The protocol aims to improve three main components: case management skills of health workers, health system infrastructure, and family and community practices. Evidence from various countries showed that IMCI can lead to improvement on under-five clinical quality of care and has the potential to reduce under-five morbidity and mortality [6-8]. Despite a substantial decline of under-five child mortality in the past five years, from 152 deaths per 1,000 live births in 2005 to 76 per 1,000 as of 2010, Rwanda still experiences a high under-five mortality [9]. This may be partly explained by gaps in IMCI implementation associated with insufficient supporting systems as well as post-training supervision and mentorship, a challenge found in other similar settings [10-12]. IMCI clinical care at HCs in Rwanda is currently supported through pre-service didactic training (lasting one week) and limited in-service supervision. The periodic supervision visits by district hospital-based supervisors are intended to provide ongoing in-service support to nurses. However, these supervisors have numerous competing demands, often leaving little time for HC visits. In addition, when supervision visits occur, they are largely consumed by data collection and reporting, limiting opportunities for on-site clinical mentoring and re-training and systems improvements.

To respond to these challenges, Partners In Health (PIH) and the Ministry of Health (MoH) in Rwanda initiated a Mentoring and Enhanced Supervision at Health Centers (MESH) program to improve quality of care [13,14]. The MESH program has two aims: 1) building the capacity of health care workers (HCWs) by bridging skills and knowledge gaps through ongoing, on-site mentoring at HCs, and 2) supporting systems-based quality improvement (QI) at HCs to address gaps in facility systems and operations [15-17].

While some evidence on the effectiveness of mentoring, particularly in infectious disease programs, has been published [15,18,19], little is known about how beneficiaries perceive and accept mentoring programs applied in the implementation of acute care programs like IMCI. The goal of this study is to explore perceptions and acceptability of MESH from the perspective of mentors, district clinical leadership and direct recipients (IMCI nurse mentees and HC directors). This study will help inform program implementers and policy makers of the key components needed and potential barriers and resistance which can be addressed proactively when implementing similar health facility-based mentorship interventions.

## Methods

### Setting

Rwandan health system has four levels of care facilities: HCs, District Hospitals, Provincial Hospitals and National Referral and University Teaching Hospitals. HCs provide a core set of basic curative services, preventative and limited inpatient care, including uncomplicated deliveries. The level of complexity and expertise increases to district hospitals with surgical and more extensive inpatient capacity to the highest level which functions as referral centers [20]. The majority of HCs are staffed by nurses who have a high school certificate in nursing sciences with few with higher level nursing degrees [21].

This study was conducted in Kirehe and Southern Kayonza districts, two rural districts in Rwanda supported by PIH and covered by the MESH program. There are 13 HCs to support 344,157 people in Kirehe and 8 supporting 194,248 in Southern Kayonza. The median distance between the district hospital and HCs is 23 km and 21 km in each district respectively and the mean distance from rural households to the nearest HC is approximately 3.5 km. Each HC has ten nurses on average, with one to three nurses trained in clinical IMCI. HC directors are all nurses by training. Their activities include management of human resources, infrastructure, and finance with limited clinical time. No HC director was an IMCI provider during the study.

### Study design

A qualitative study using focus group discussions (FGDs) and in-depth interviews was conducted to investigate perceptions of the MESH program as delivered as well as acceptability and perceived benefits of the MESH program from HCW mentees providing IMCI services, HC directors, and district hospital directors. All 21 HCs in Kirehe and Southern Kayonza were included. The study took place from January-March 2012.

### The MESH intervention

The MESH program was integrated within the existing District Hospital supervisory system and is described in detail elsewhere [15]. MESH mentors are Rwandan nurses with a university nursing degree and hands-on experience in their clinical area. Mentors were selected based on competency in clinical domains (written examination) and experience and competency in mentoring or coaching and interpersonal skills (interviews). These followed national hiring procedures and incorporated World Health Organization (WHO) clinical mentoring guidelines [19].

Mentors make daily mentoring visits to HCs, providing side-by-side mentoring, coaching and support to HCWs in translating data into quality improvement initiatives.

The MESH model adds several enhancements to the standard approach to implementing IMCI. First, the MESH program decentralizes pre-service training by offering training at district health facilities. Second, the district supervisory structure is augmented by MESH supervisors, who focus primarily on clinical mentoring of nurses and quality improvement of HC care. These supervisors make routine 2–3 day intensive visits to HCs every 4 to 6 weeks, during which they provide on-site case management observation and side-by-side mentoring, support for higher-level problem solving, diagnostic and decision-making skills, lead case discussions, and help the HC team address quality issues. The clinical supervisors also work to strengthen the availability of performance data to feedback improvement by routinely capturing data on nurses’ clinical skills, facility conditions, and clinical indicators which was used for real-time feedback to the HCs and also contributed to a district-wide monitoring, evaluation and QI system [22].

### Data collection

Two focus group discussions with IMCI HCWs and two focus groups discussions with HC directors from two MESH-supported districts, for a total of four FGDs (Table 1). HCWs were eligible if they had received at least three mentoring visits by a MESH mentor in the last six months. When more than one HCW at a facility was eligible, the one who had received the most clinical mentorship was selected. In addition to FGDs, in-depth interviews were conducted with the district medical director and the IMCI mentor from each district (see Additional files 1,2,3 for FGD and interview guides). Table 1 summarizes characteristics of study participants.

**Table 1:** Focus group discussion and interview participant characteristics

**Participants by district**	**Level of education**	**Number of included participants**	**Average professional experience in years**	**Average mentoring visits received within last 12 months**	**Type of data collection**
Kirehe district					
Nurses	A2^¥^	9	5.5	12	FGD
	A1^§^	2	2.3	12	
Health center directors	A2	12	6.2	12	FGD
	A1	1	5.3	10	
IMCI mentor	A1	1	5	N/A	Interview
District medical director	A0^ǁ^	1	>15	N/A	Interview
Southern Kayonza district					
Nurses	A2	6	5.7	14	FGD
	A1	2	2.8	15	
Health center directors	A2	7	7.1	10	FGD
	A1	1	3.2	10	
IMCI mentor	A0	1	6	N/A	Interview
District medical director	A0	1	5	N/A	Interview

^¥^High school (secondary) level as defined by Rwanda education council.

^§^Two to three years of post-secondary education as defined by Rwanda education council.

^ǁ^Bachelors degree as defined by Rwanda high education council.

An external, independent moderator facilitated discussions in Kinyarwanda while a note-taker recorded the session using a digital recorder and writing notes. A set of open-ended questions were prepared to guide the interviews and FGDs. Each item was followed by probing questions. The guide was pilot tested with IMCI nurses who had been mentored but who were not included in the study. Data from field testing was analyzed and informed further adaptations of the guide as well as creation of new codes.

Interviews and FGDs took place in non-clinical settings to ensure that providers were not called away to provide clinical care. Participants were assigned a number depending on their seat locations in the FGD and no personal identifiers were recorded. The interviews varied between 60–70 minutes long, while FGDs took between 60–90 minutes. All audio tapes were transcribed verbatim and translated into English.

For quality assurance, 10% of pages (selected randomly) from each English transcript was “back translated” to Kinyarwanda. The back translation was performed by a second translator and was compared to the original Kinyarwanda transcript. As no important differences in meaning were noted, the translation was determined to be of sufficient quality to proceed with analysis.

### Data analysis

Four steps were followed during the data analysis: 1) immediate debriefing between data collectors and study Principal Investigator (AM) after each focus group, 2) listening to the tape and transcribing the content of the tape, 3) checking the content of the tape with the moderator and noting any non-verbal behavior, and 4) back translation of a sample prior data analysis, as noted above [23]. The analysis team included the MESH program management team and experienced qualitative data analysts from the National University of Rwanda School of Public Health and from Partners In Health, Boston.

All transcripts were saved as word documents and were imported into Atlas.ti v5.2 for analysis. The aim of the analysis was to bring to light an underlying sense of the transcripts [24,25]. A list of codes reflecting specific study objectives was pre-defined by study investigators (see Additional files 4-5 for pre-defined codes). This list was not exhaustive and additional codes were added iteratively throughout several readings of the transcripts [26]. The hermeneutic analysis consisted of linking themes to developed codes [27-30], thereby capturing and organizing the main themes and ideas shared during the FGDs and interviews.

### Ethical considerations

Confidentiality of responses was strictly maintained. The moderators of FGDs and in-depth interviews were independent from the MESH program with no vested interest in the responses given by participants. Written, informed consent was obtained from all participants. A clear statement introducing the purpose of the study, ground rules, language, and participants’ questions were addressed by the moderator prior to data collection. The study was reviewed and approved by Rwanda National Ethics Committee and the Partners Institutional Review Board in Boston, Massachusetts.

## Results

Forty-four health workers from Kirehe and Southern Kayonza districts participated, including 40 direct HCW recipients of MESH support, two district hospital directors, and two IMCI clinical mentors (Table 1). Five themes were identified as participants’ perceptions regarding acceptability and benefits of the MESH program, including 1) interactive, collaborative capacity-building, 2) active listening and relationships, 3) supporting not policing, 4) systems improvement and 5) real-time feedback. Three themes – interactive, collaborative capacity-building active listening and relationships, and systems improvement – were pre-identified. The two themes of supporting not policing and real-time feedback emerged during FGDs and new codes were developed. Staff turn-over, insufficient infrastructure, drug stock-outs and supply shortages were highlighted as challenges and targets for future improvement of the MESH program.

### Positive attributes of MESH program design

#### Interactive, collaborative capacity-building

Respondents focused on the unique approach mentors took to their work on skills assessment and building. This interactive and collaborative capacity-building was perceived as an approach to build their confidence. For example, one IMCI provider supported by the rest of providers mentioned:

“They built our confidence not only in IMCI case management but also in general nursing care we provide every day. I feel proud of the work when I can handle even the complicated cases that I could not treat before due to their support.”

They provided many anecdotes expanding on this the interactive process that MESH mentors used to assess their mentees’ skills and then help build their capacity based on their findings through a supportive learning process. These focused on mentors who provided side-by-side mentoring, identifying areas in need of improvement, and then targeted support and guidance on new updates to IMCI protocols and case management. For example, as one of the IMCI providers expressed:

There are often times that you find it difficult to use the new protocol because you don’t have enough knowledge on it. Mentors come to help you to learn. Another example is on malnutrition and HIV classification and treatment which got integrated into IMCI guideline recently and mentors taught us about classifying and managing those cases.

These responses suggest that respondents perceive that MESH program facilitates the enactment of appropriate solutions and new protocols through a responsive process between mentors and mentees, an attribute which was important in acceptability of and benefits from the program.

### Active listening and relationships

Most of the FGD participants also noted the importance of active listening as a key component of relationship-building between mentors and mentees, an important factor associated with acceptability and perceived benefits. The trust formed through these established relationships was described as improving mentees’ openness to learning. For example, one IMCI provider stated:

Well, to be able to trust [in] somebody, one has to be close to you and you are able to learn from them. I may consider you as somebody who is knowledgeable, but the way you conduct yourself may mean that I cannot be able to ask you all the questions that I may have. Mentors are humble and speak with us.

As exemplified by the quotation above, participants viewed active listening and humility as fundamental elements critical to building trust and productive mentor-clinician relationships. Here “humility” was described as one of qualities of mentors, that consists in a non-judgmental consideration of HCW mentees.

### Supporting not policing

The use of supportive methods to encourage change and improvement was identified while exploring the difference between clinical mentoring and the classic style of supervision used prior to the implementation of the MESH program. This was important in contributing to the perceived benefits. Reinforced by most participants nodding heads in agreement, one FGD participant described the difference:

With the old system, the supervisors used to come to the health center, and they would suspect you and try to capture what you are not doing correctly. They were scaring you. Also, supervisors did not have enough time to stay around and to get used to you or vice versa. But with mentoring, even though I can say that the time they have is not also enough, but they have much more time to spend with us.

This response also highlighted that MESH mentees felt that the old supervision system tended to put clinicians on the defensive and allowed less time for productive mentorship and learning. In contrast, they felt that the MESH program allowed the mentors and mentees to take better advantage of the limited amount of time available.

### Systems improvement

In addition to technical support that mentors provide, respondents perceived that mentors provide valuable assistance in improving and strengthening systems. As one HC director stated:

….They [mentors] helped me to build a system. Before they start visiting health centers we had two bad and routine practices: 1) Children under five were on same queue as adult patients. We did not have a system to triage under-five or any other severe cases 2) IMCI clinic was not working every day because we did not understand why having IMCI and adult consultations the same day. Now IMCI clinic is always running every day and ready to receive each under-five without waiting too long. Also, mentors helped to establish a triage system where a nurse makes a quick assessment of severe cases on queue and get them into concentration room first.

Respondents frequently gave specific examples to show how MESH helped them replace “bad” practices with stronger routine systems that allow them provide better and more efficient care.

One HC director from Kirehe district described how a mentor helped improve the HC’s staff scheduling system:

…I did not care about the importance of scheduling my staff according to their training background. Before mentors visited my health center, I would schedule nurses in whatever service regardless their expertise and/or limitations. It made IMCI clinic less functional, working mostly once or twice a week--but since they [mentors] came to my clinic, we had a plan together to improve my schedule so that IMCI clinic may be open every day. Now, it is running very well every day from Monday to Friday and all children under-five are treated using IMCI.

### Real-time feedback

Real-time feedback was defined by respondents as the prompt reaction of mentors to correct and guide mentees during or immediately after an IMCI consultation, also important in making the program more acceptable and helpful. Mentors described how they provided constructive criticism on the IMCI mentees’ decision-making processes during their observation sessions and on the HC systems supporting IMCI at the time of their visits. This kind of immediate feedback was highly appreciated by mentees, directors of HCs and district hospitals, and they described it as a key beneficial strategy of MESH for addressing their challenges in classification and treatment. The use of this effective feedback transforms capacity in knowledge, skills and practice to HCWs was one of the core instruments of MESH to improve quality of care. Respondents described how this improvement occurred due to the immediate and non-judgemental correction of a mistake or of a skipped step when observing a child’s clinical encounter, as well as more general feedback mentors provide at the end of a mentorship or during the follow up mentorship visit.

For example, one IMCI provider mentee mentioned:

… he [mentor] is patient, and does not bear a grudge against nurses who are inflexible. An example is when he found out that I had not implemented his previous recommendation on systematic vital signs to all children. When they came back to health centers he gave the same feedback. He never gets tired of talking or showing how to improve IMCI consultations.

Respondents also described the importance of higher-level feedback the mentors gave at periodic district meetings. One HC director noted that monthly district-based debriefing meetings were seen as a valuable feedback opportunity: “There is a meeting that we have monthly and [mentors] show what they saw was going well and what was going bad at the HCs and together we discuss strategies to fix gaps”.

### Barriers to the MESH program

Several areas were identified as barriers to IMCI implementation and the MESH program’s support of IMCI implementation. Staff turn-over was one concern raised by many participants. Nurses were drawn to other HCs for better wages and preferred location, a challenge that district leadership reported not able to overcome. As a result, the increased individual provider capacity resulting from MESH was sometimes lost to the HC.

Additionally, IMCI mentors and provider mentees highlighted infrastructure as a barrier to MESH and IMCI. Infrastructure-related challenges included insufficient number or small size of consultation rooms and lack of or inappropriate materials and medical facilities. Discussing the effects of infrastructure constraints, one mentor said:

Some health centers were constructed so many years ago, and so they have very little or not appropriate facilities. In some health facilities, [they] have only one consultation room accommodating adult and pediatric cases. This makes it hard to do mentorship to IMCI nurses because nurses are always obliged to do a mixed consultation including adult and pediatric cases.

While many noted drug stock-outs and supply shortages as a challenge to IMCI implementation, one district medical director also noted this as a barrier to clinical mentoring process itself.

When the district pharmacy lacked basic medications, this limited the ability of health care workers to implement new skills and knowledge gained during the mentoring process…for example, they would learn to prescribe a right drug but when that is not available, it becomes impossible to apply their new skills.

In response, stock-outs at the health facility level were an area of focus of MESH program activities, and mentors highlighted the reduction of health facility stock-outs as one of their targets in supporting systems improvement during their mentoring visits. One mentor noted:

“I feel amazed to have reduced frequency of stock outs. No more complaints from nurses about stock-outs and no more children gets sent back home due to lack of essential drugs”.

### Recommended improvements to the MESH program

Three strategies were frequently mentioned when discussing approaches that mentors should use for HC support in addition to the on-site mentoring: on-site teaching sessions (slide presentations), case discussions and phone calls for ongoing support in-between visits. Some HCWs had received an outdated IMCI training and suggested general IMCI refresher trainings. Reflecting the ongoing learning within MESH, clinical mentors had already incorporated IMCI updates using adult learning and other methods including case discussions into their clinical mentoring visits, with one mentor stating:

“I found a combination of various techniques very effective but one condition has to be respected: Involve providers in the learning process, let them ask questions, observe and give them a time to practice to implement your feedback.”

Another suggested improvement was for mentors to be trained in different spheres so that they could provide a comprehensive package at HCs, rather than focusing on one clinical domain. One district hospital director said:

Trainings in all domains should be given to these workers so that they may not feel limited when they are at health centers. For example, a mentor should not go to the clinic for only IMCI. He should also feel comfortable in supporting other services like maternity and others. They should provide the minimum package that health centers provide. If this happened, they would go health centers and spend even five days and ensure that health center got all trainings needed. Also, this would reduce the cost related to inviting HCWs to attend trainings. If each district had at least five mentors, it would be a good number that can improve skills and knowledge at health center.

### Acceptability

When asked about the acceptability of the MESH program, all respondents expressed their desire to continue the program as a strategy to maintain high quality pediatric care. One mentor described the value of the mentorship process by using a concrete example:

I do support that MESH continues this support because you can find that, for example, antibiotics were inappropriately prescribed for children without respiration problems. …the incidence of wrong use of drugs has gone down. The nurses only give drugs to patients who really need them.

The district clinical leadership and IMCI providers expressed strong acceptance of the MESH model, and advised that the program be expanded. As one of district medical directors said:

I would recommend that MESH continues working in this district and in fact have more support for it. In more support, I mean having more staff, first of all, [and] secondly, having them spend more time at the health centers. … [In some cases,] they should have their own car so that they can be able to reach the health centers early enough.

One IMCI provider [colleagues nodding heads to support her argument] mentioned “…there [are] three main reasons why MESH should continue: It builds our ability to classify and treat children, increased our self-esteem and confidence, and mentors are always respectful to us.”

## Discussion

The results of this study demonstrate strong support for the MESH program by IMCI health care workers, mentors and district clinical leadership. This support reflected the perceived benefits as well as the acceptability of the approach. Health care workers considered the clinical mentoring to be a strategy that built their capacity. This kind of ongoing capacity building is especially important, given that most health care workers in rural and resource-limited settings have relatively limited formal education and very limited continuous skill-building opportunities [31].

We found that the combined clinical mentoring and QI approach focusing at the individual provider and HC levels was not only acceptable, but also offered considerable benefits. Health care workers highlighted positive perceptions of the benefits and critical components and approaches of the MESH program that contributed to its perceived success. These included methods of knowledge transfer, mentoring characteristics such as active listening and real-time feedback, and integration of support for systems QI. The study complements results from a quantitative evaluation that demonstrated improvements in IMCI care in a variety of domains two years after the MESH program implementation. These included 1) increase in IMCI services availability (daily and by IMCI-trained nurse); 2) enhanced adherence to current clinical protocols, and 3) improved classification of the severity of illness of patients [15].

Consistent with findings from other studies, we found that clinical mentoring strengthens HCWs’ self-reported knowledge, perceptions and confidence [32-34], enables orientation of less experienced HCWs [35], promotes staff satisfaction and retention [36,37], and reinforces implementation of IMCI quality of care in rural and limited-resource settings [38-40]. Furthermore, mentorship skills in active listening, humility, and non-judgmental feedback created mentor-mentee relationships that contributed to effective teaching and coaching of HCWs with low levels of education and minimal experience in IMCI. Hill and Sawatzky [41] also described these characteristics as key for optimal transformation of nursing practice [41]. A core component of training the mentors was in these interpersonal skills, in addition to the clinical technical ones.

As key components of clinical mentoring interactions, this study suggests that mentoring skills of active listening and relationship-building play an important role in transfer of clinical capacity and build morale, confidence and self-esteem of both the mentor and HCW mentee. Study participants highlighted mentorship as a form of technical assistance that district hospitals should adopt to support HCs. Active listening and relationship-building were linked to the ability of mentors to achieve buy-in from their mentees on a wide range of challenges, from limitations in mentee knowledge and practices to systems issues, such as staffing schedules and facility-level supply chain management.

The clinical mentorship provided through the MESH program was also perceived differently from the traditional supervision system. The traditional system is considered more punitive and not on supportive mentorship. Typical supervisors were perceived as “police agents,” rather than technical facilitators and indeed, in many countries, traditional supervision has been criticized by clinicians as an ineffective approach [42]. In contrast, the MESH program was perceived as a program that facilitates supportive engagement between mentors and mentees and fosters collaborative quality improvement. Using this approach, MESH mentors have more time to spend directly with health care workers, rather than on monitoring and reporting.

Not surprisingly, a number of challenges existed in optimizing the benefits of the MESH program. Staff turnover, a challenge across resource limited settings [43], was demonstrated as a handicap of the MESH intervention. When mentored HCWs left the facilities, it required much time and effort to find replacements and to help the replacements achieve the same skill level as their mentored predecessors. The unplanned and frequent turnover created a need for more frequent clinical mentoring visits to HCs. However, mentorship also provided additional needed support for new nurses who may not have had access to repeated formal didactic trainings prior to starting providing care.

Frequent stock-outs limit the ability of HCWs to prescribe appropriate medications and reduce the quality of care despite providers’ knowledge and skills of IMCI protocols [44,45]. Lack of equipment and stock-outs were both threats and common challenges to clinical mentoring. Some HCs without basic equipment could not implement recommendations from clinical mentoring visits. This study showed that IMCI clinical mentors were seeking to address facility-level systems challenges that led to stock-outs, but there was also a need to improve these systems at a district or national level. Similarly, infrastructure and systems issues were highlighted both as handicap to implementation and as targets for improvement through the MESH intervention.

This study has a number of limitations. Only providers who were trained in IMCI and who received MESH mentoring were included. Therefore, the generalizability of our findings to health care workers not trained in IMCI and other settings is limited. In addition, some participants may have been hesitant to share negative experiences about the MESH mentors. We believe that using experienced external data collectors has minimized this effect. This study focused on assessing HCWs’ perceptions and the acceptability of the MESH program. Further study to understand the effects of the MESH intervention on patients’ perceptions of quality of care would be valuable. Studies of the cost-effectiveness and scalability of MESH are underway and will add to understanding of scalability and sustainability of the MESH model.

## Conclusion

This study is consistent with other research and recommendations regarding clinical mentoring interventions as an effective strategy to build capacity of health care workers and improve the quality of care at HC level in developing countries [46]. We found positive perceptions and strong acceptance of the MESH clinical mentoring intervention by nurse mentees, mentors, and HC directors. Reasons for positive perceptions and acceptability of the MESH program varied between the different types of respondents: while directors mainly linked their positive perceptions to the role of mentors in improving specific health systems, IMCI mentees and mentors spent more time discussing the collaborative capacity-building, active listening and relationship-building process as the most positive aspects of the MESH program.

Although systems and operations issues were limiting factors to clinical mentoring program implementation, the MESH model is promising. Tackling these limitations could facilitate effective implementation of IMCI mentorship program with a goal of improving clinical outcomes. Increasing the number of mentors and building their capacity through training to enable them to cover more than one clinical domain is a potential area to be explored.

## Competing interests

The authors declare that they have no competing interest.

## Authors’ contributions

AM designed the study, recruited study participants, monitored data collection, carried out data analysis and drafted the manuscript. HM and FRC reviewed the study design and edited the manuscript. APM and LN provided technical support in study protocol development, and edited the manuscript. BHG, LRH and JN supervised the study, data analysis and manuscript development. All authors provided critical revision of subsequent drafts and read and approved the final manuscript.

## Authors’ information

Authors Lisa R Hirschhorn and Joseph Ntaganira are co-senior authors.

## Pre-publication history

The pre-publication history for this paper can be accessed here:

http://www.biomedcentral.com/1472-6963/14/275/prepub

## Supplementary Material

Additional file 1 IDI guide-IMCI MESH mentors. Click here for file

Additional file 2 FGD guide-IMCI Nurse mentees. Click here for file

Additional file 3 IDI guide-district Medical Director. Click here for file

Additional file 4 Codebook-Nurse mentees. Click here for file

Additional file 5 Codebook-Others. Click here for file
