# Supplementary material for: Clinical mentorship to improve pediatric quality of care at the health centers in rural Rwanda: a qualitative study of perceptions and acceptability of health care workers
Source: BMC Health Serv Res. 2014 Jun 20;14:275. doi: 10.1186/1472-6963-14-275 (PMC4077561; doi:10.1186/1472-6963-14-275)
Supplement: Additional file 1 — IDI guide-IMCI MESH mentors. [file 1472-6963-14-275-S1.pdf]

Focus Group Discussion Guide: Nurse Mentees

Code	Definition
**1. IMCI definition**	
1.1. Care and treatment	Integrated or comprehensive management of childhood illness(below the age of five)
1.2. Satisfaction	Satisfy the needs of the mother who brings in their child by giving a correct treatment
1.3. Counseling	Advice to mothers about their child's life, feeding practices or their own reproductive health
**2. MESH description**	
2.1. Friends	Working together at health centers, humble, receptive making corrections without reprehending.
2.2. Reminders	Help out and remind them when something cases are difficult to handle
2.3. Trainers	Inform there are latest changes in the IMCI guidelines, and/or things that didn't change
2.4. Improve quality	Observing the actual quality of care and observe challenges and help to improve
2.5. Exchange	Discuss on different ideas and get inputs. Consider ideas my ideas as valuable too.
2.6. Knowledge	Improve some one's knowledge and intellectual capacity
2.7. Feedback	Summarizing or telling what when well and what went bad and show what has to be done and how and encouraging to continue doing well
2.8. Keep contact	Keep the correspondence on complicated cases even during the time there is no physical presence. Through the phone
2.9. Refresher training	Need to re-training and provide updates on new protocols
2.10. Equipment and supplies	Direct provision of materials or advocacy to equip health centers including registers, case management recording forms, medications etc.
**3. Communication& Skill-Building**	
3.1. Knowledge assessment	Ability to discover where needs a support./gaps through observation and checklists
3.2. Support	Helping or providing support to manage complicated cases by helping or responding to difficult questions
3.3. Relationship	Agreeing and accepting when there are difficult situation. Talk to people and care on what they tell him
3.4. Paperwork	Difficulties due to different registers and report to be filled out
3.5. Dosing tools	Inconsistency of dosing materials; especially spoons
3.6. Patient flow	An increase of the number of patients associated with difficulties to do any more activities
3.7. Customer care	Ability to interact with client, essentially marked by saluting and do an identification before anything else
3.8. Comfortable	Possibility to speak and/or ask question easily when is necessary
3.9. Respect	Answer questions and collaborate without undermining

3.10.Scare	Cause fear to someone.<br>Intimidating or reprimanding, terrorizing
3.11.Special	Particular aspects that mentors have that is unique to them. Which you can't get with traditional supervision
3.12.Introduction	Description of personal identification humbly and comprehensively. Mentioning your role without imposing
3.13.Schedule sharing	Gaps in sharing schedules prior the mentoring visit
3.14.Active listening	Good listening and supported by follow up
3.15.Positive perception	A neutral judgment especially even when you would be in a mistake
4. **Mentoring vs.traditional supervision**	
4.1. Bad collaboration	Clinical IMCI and community IMCI. This reflect a area of improvement
4.2. Missing appointment	Children who don't show up while he/she or the caregiver got a rendez-vous to get back to the health center
4.3. Inspection	Reaching health centers and observe what is done well or bad having intention to reprimand rather than supporting and coaching
4.4. Projection	Using projector and videos to train and demonstrate danger signs and management of cases
**5. Barriers to IMCI delivery & MESH contributions**	
5.1. Initial training	Need of didactic trainings in IMCI to fill the gaps
5.2. Refresher training	A training aiming to provide updates on IMCI protocols and/guidelines
5.3. Organization and Scheduling	Organizational problem that makes IMCI less effective especially in deploying nurses. Misalignment between training background and clinical assignment
5.4. Paperwork and reporting	All constraints related to the length of forms filled out by nurses and other clinical reports that nurses are required to fill out
5.5. Misalignment	Discordance between clinical responsibility and training background. IMCI trained nurses appointed other tasks other than IMCI consults
5.6. Stock out	Lack of one or more essential IMCI medicine at both health center and district pharmacy
5.7. Attrition	Trained personnel leave to other provinces or countries looking for access to good wages and other reasons of lack of motivation
5.8. IMCI rooms	Consultation room availability at health centers
5.9. Infrastructure	Problem with IMCI related equipment and infrastructure such as rooms etc.
5.10.Protocol and patient charts	Sources and effort to supply equipment to health center IMCI clinics
5.11.Active IMCI clinic	A clinic running at least 5 days per week despite other challenges mentioned
5.12.Education level	Low education level, most of IMCI trained nurses are A2 which limits them to further understanding limiting mentoring effectiveness
5.13.Academ_deviation	Nurses always like to continue with their studies and decide to study whatever they want (other than nursing)
5.14.Performance-based financing	All reasons related to incentives provided by the ministry of health
5.15.IMCI implementation	MESH program facilitated the implementation of IMCI at health centers. This is an implementation that was delayed for different reasons. Example: No IMCI clinical despite the initial training that hc nurses had gotten.

5.16. Assessment	Whatever activities to assess gaps leading to setting potential intervention to improve IMCI quality of care at health centers
**6. Acceptability and expansion**	
1.1. Acceptability and expansion	Argument or reaction to support MESH program acceptability and expansion
