# Supplementary material for: Clinical mentorship to improve pediatric quality of care at the health centers in rural Rwanda: a qualitative study of perceptions and acceptability of health care workers
Source: BMC Health Serv Res. 2014 Jun 20;14:275. doi: 10.1186/1472-6963-14-275 (PMC4077561; doi:10.1186/1472-6963-14-275)
Supplement: Additional file 2 — FGD guide-IMCI Nurse mentees. [file 1472-6963-14-275-S2.pdf]

Focus Group Discussion Guide: Others

Code	Definition
**1. IMCI definition**	
**1.1. IMCI def**	All responses viewpoints referring to the explanation of IMCI including U-5 care, assessment of danger signs, guide to correct classification and treatment
**2. MESH description**	
2.1. MESH def	All responses or viewpoints to define or describe MESH program such as side by side coaching, supportive supervision and capacity transfer. This doesn't include detailed MESH structure or activities coded in section 3 and 4
**3. Communication& Skill-Building**	
3.1. Knowledge assessment	All information about ability to discover where needs a support./gaps through observation and other techniques during mentoring visits
3.2. Support	All information about the package provided by mentors during their visits at health centers this includes detailed package that mentors offer to nurse mentees
3.3. Relationship	Whatever expression of connection between mentors and mentees. These include, feeling, living along and flexibility
3.4. Paperwork	All information about the paper work explained as an addition work to care providers
3.5. Patient flow	All difficulties related to increase of patient volume at IMCI clinics
3.6. Customer care	All positive improvement on provider and client interaction and communication. This code will captures their own appreciations or positive change in receiving and communicating with clients
3.7. Respect	All reactions or behavior responses revealing a respectful behavior or a sense of collaboration without undermining. This applies only to mentor-mentees
3.8. Schedule sharing	All information or wish to share schedule of planned health center mentoring visit
3.9. Active listening	All information related to attentive and non judgmental listening. This code is applicable only between mentor and mentees
4. **Mentoring vs.traditional supervision**	
4.1. Ment.positive	All positive arguments about mentorship model on one or more activities
4.2. Ment.negative	All negative arguments about mentorship model on one or more activities
4.3. Supervision negative	All negative arguments about supervision model on one or more of its components
4.4. Supervision positive	All positive reactions or arguments about supervision system
4.5. No difference	All statements confirming a similarity of both mentoring and supervision
**5. Barriers to IMCI delivery & MESH contributions**	
5.1. Training	This code stands for needs and others related training information expressed as a barriers to IMCI delivery
5.2. Staffing	All challenges in IMCI delivery due to misalignment between training background and clinical assignment
5.3. Paperwork and	All constraints related to the length and number of forms to be filled out by

reporting	nurses and other clinical reports that nurses are required to complete on daily basis
5.4. Stock out	All information related to lack of one or more essential IMCI drugs and supplies at both health center and/or district level
5.5. Turn over	All information related attrition of nurses to other provinces or countries
5.6. Infrastructure	Information related to equipments and infrastructure such as consultation rooms and other facility and system
5.7. Protocol and charts	All information related to patients and charts and protocols supply chain expressed as a challenge to IMCI quality of care
5.8. Education	Information related to formal education program, levels, and package mentioned as a challenge to IMCI implementation
5.9. Performance-based financing	All reasons related to incentives provided by the ministry of health
5.10. IMCI implemention	This code stands for all information related to MESH contribution to overcome challenges mentioned in code 5.1-5.9
**6. Acceptability**	
6.1. Acceptability	All arguments or reaction to support MESH program acceptability and expansion
