# Supplementary material for: Clinical mentorship to improve pediatric quality of care at the health centers in rural Rwanda: a qualitative study of perceptions and acceptability of health care workers
Source: BMC Health Serv Res. 2014 Jun 20;14:275. doi: 10.1186/1472-6963-14-275 (PMC4077561; doi:10.1186/1472-6963-14-275)
Supplement: Additional file 3 — IDI guide-district Medical Director. [file 1472-6963-14-275-S3.pdf]

Focus Group Discussion Guide: Nurse Mentees

Introductory Script:

Thank you all for taking part in this focus group discussion. The purpose of this discussion is to learn your thoughts about what is working well with the MESH mentoring program and IMCI, and also how this program could be improved.

So that we can keep track of what people are saying, remember that only one person should be talking at a time. Please do not interrupt someone when they are talking.

Everything you say today will be kept completely anonymous and confidential: this means that we will not share your name with anyone or let anyone know which ideas you agreed or disagreed with.

We hope you will be comfortable giving us your honest opinions, even when you disagree with what other people are saying: there are no wrong answers, and we can learn a lot from the unique experiences that each of you have had.

This discussion will take one to two hours. At the end of this session, we will provide food, refreshment, and mean of transport to all participants.

Are there any questions before we begin the discussion?

Iriburiro

Tubashimiye gufata umwanya wanyu ngo mugire uruhare muri iki kiganiro.Impamvu y'iki kiganiro ni ukukusanya ibitekerezo kubijyanye n'imikorere ya program ya MESH na PCIME, kandi tukanamenya uko twanoza imikorere yiyi gahunda.

Ibi biradufasha kumenya uko uko abantu bavuga kuri iyi gahunda.
Tubibutse hagenda havuga umwe umwe tworonda ko harira uca undi mu ijambo.
Ibyo muri buvuge byose uyu munsi bizabikwa mu ibanga: ibi bivuga ko tuta zerekana amazina yanyu n'uwariwe wese cyangwa tutazatangaza ibitekerezo buri wese yatanze yemeza cyangwa ahakana zimwe mungingo z'iki kiganiro.

Turizera ku muri bugire kwizanzura igihe mutanga ibitekerezo byanyu,nubwi mwaba mutemeranya n'ibyo abandi bavuga:nta gisubizo gipfuye, kandi dushoobora kwigira ku bunararibonye bwa buri wese.

Iki kiganiro kiratwara isaha imwe kugera kuri ebyiri. Nyuma yacyo, turafata ifunguro,n'ikinyobwo ndetse n'amafranga y'urugendo kubitabiriye iki kioganiro bose.

Haba hari ibibazo mbere y'uko dutangira ikiganiro?

Setting the Stage

1. In your own words, what is IMCI?

   Mbese mukoresheje amagambo yanyu, PECIME ni iki?

   a. What specific IMCI services are offered to sick children at this health center?
      Ni izihe servisi za PECIME zihabwa abana barwaye kuri iki kigo Nderabuzima?

2. How would you describe what the MESH program is?

   Ni gute mwasobanura program ya MESH icyo ari cyo?

3. Remember the last visits you experienced with a MESH mentor. What types of activities did the mentor do with you?

   Ibuka ku gihe kimwe mwakoranye n'umugenzuzi uhugura(mentor)wo muri MESH. Mwakoranye iki?

   a. How do IMCI mentors share their clinical knowledge during mentoring visits?

      Ni gute umu aba mentors babaha ku bumenyi bafite mu buvuzi mugihe baje mukazi kabo k'ibugenzuzi no guhugura ku kigo nderabuzima?

   b. How do they give you feedback about what you are doing well or what you could improve?

      Ni gute baha abaforomo ibitekerezo kubijyanye n'ibyo bakora cyangwa ibyo bakwiriye kuvugurura

   a. How could the mentor have improved their support to you during their last visit?

      Ni gute umugenzuzi uhugura (mentor) yagombaga kuba yarafashije muburyo buruseho kuba bwiza igihe aheruka kubasura?

<u>Communication& Skill-Building</u>

4. Please tell me about a time when you did not understand something that the mentor was trying to explain.

   Ndifuza ko mwampa urugero rw'igihe mutumvise neza ibyo umugenzuzi uhugura(mentor) yageragezaga kubigisha.

   a. What did you say to the mentor when you did not understand?

      Niki mwaba mwaramubwiye igihe mutumvise neza?

      - Did you ask the mentor to repeat or explain in a different way?
      - *Mwaba mwaramusabye gusubiramo cyangwa kubisobanura mubundi buryo?*

   b. How did the mentor respond to you when this happened?

      Ni gute yaba yarabasubije?

5. What skills have you personally gotten from mentors?

   Ni ubuhe bumenyi mwe ubwanyu mwaba mwarungutse, bitewe no gukorana n'umugenzuzi uhugura (mentor)?

   a. Do you value these skills? How so?

      Mubona hari akamaro bufite?Mubuhe buryo?

   b. Are there ways that these skills are <u>not</u> helpful to you, or could be better?

      Haba hari uburyo mubona ubwo bumenyi butabafitiye akamaro?cyangwa bwarushaho kunonosorwa?

<u>Respect : Ikinyabupfura</u>

6. Do you feel respected by mentors?

Mbese mwumva mwubashwe n'abagenzuzi bahugura?

a. How does the mentor express respect during his/her visits?

   Mbese ni gute n'umugenzuzi uhugura agaragaza ikinyabupfura mugihe yaje mukazi ku kigo Nderabuzima?

b. Are there ways the mentor could be more respectful? How so?

   Hari icyo umugenzuzi uhugura yakora cyatuma mwumva mwubashwe biruseho?Mu buhe buryo?

<u>Trust: Icyizere</u>

7. How do you feel in asking mentors questions about new concepts or challenges you are experiencing?

   Mbese mwumva mumeze mute iyo mubaza umugenzuzi uhugura ibibazo bijyanye n'imikorere mishya cyangwa imbogamizi mwaba muhura nazo?

   a. How does the mentor react when you ask questions?

      Iyo mumubajije ibibazo abyifatamo ate?

   b. In your experience, are mentors good listeners? How so? How could they improve?

      Ukurikije ubunararibonye bwawe, ubona abagenzuzi bahugura bategа amatwi neza? Ni gute banoza gutega amatwi kwabo?

8. How comfortable do you feel admitting to the mentor when there is something you don't know, or can't remember?

   Ni gute mwumva mwisanzuye kwegera umugenzuzi uhugura igihe hari ikintu mutazi cyangwa mutibuka?

   a. Is there anything that would make it easier for you to share your challenges with the mentor?

   Haba hari icyatuma mwumva biboroheye/mwisanzuye kubwira umugenzuzi uhugura ibibazo mufite?

<u>Mentoring vs.traditional supervision:</u> *Ubugenzuzi mpugura n'ubugenzu busanzwe*

9. How can you describe the difference between the roles of clinical mentors compared to traditional supervisors? Particularly in IMCI?

   Ni gute mwasobanura itandukaniro riri hagati y'inshingano z'abagenzuzi bahugura n'umugenzuzi basanzwe(muburyo bwari bumenyerewe kera)? Cyane mwibande kuri PCIME?

   a. In which ways is mentoring different from traditional supervision?

      Ni mubuhe buryo kwigisha uhugura (mentoring) bitandukanye n'ubugenzuzi busanzwe (muburyo bwa kera)

   b. In which ways are mentoring and traditional supervision similar?

      Ni mu buhe buryo kwigisha uhugura(encadrement) n'ubugenzuzi busanzwe byaba ari bimwe ?

<u>Areas of improvement Ahakwiriye inonosorwa</u>

10. What components of mentoring do you think need improvement?

Ni ibihe bice bya gahunda y'iyigisha uhugura(mentoring)mutekerezako byaba bikeneye kunozwa?

11. What changes would you suggest to improve IMCI MESH program health centers?

    Ni izihe mpinduka wifuza zarushaho kunoza imikorere ya programu ya MESH muri PCIME ku bigo nderabuzima?

    c. Changes to mentoring visit structure?

    Impinduka mubijyanye n'uko isurwa rikorwa?

    d. Knowledge or skills of mentors?

    Ubuhanga n'ubumenyi bw'abagenzuzi bahugura

    e. Mentoring techniques?

    Ibijyanye na tekiniki bakoresha muguhugura no kwigiisha?

    f. Other?

    Ibindi?

<u>Barriers to IMCI delivery & MESH contributions Imbogamizi muri gahunda ya PECIME n'uruhare rwa MESH</u>

12. What barriers do you find to IMCI delivery?

    Ni izihe mbogamizi muhura nazo muri gahunda ya PCIME?

    a. Nurse training and skills?

    Imbogamizi zijyanye n'ubumenyi bw'abaforomo n'amahugurwa?

    b. Systems for nurse assignments and clinic scheduling?

    Imbogamizi zijyanye n'imikorere y'ikigonderabuzima n'uburyo akazi gapangwa

    c. Paperwork and reporting challenges?

    Imbogamizi zijyanye no kuzuza impapuro n'amaraporo?

    d. Medication and equipment availability?

    Ukuboneka kw'imiti n'ibikoresho nkenerwa?

    e. MOH support?

    Ubufasha butangwa na ministeri y'ubuzima?

    f. Performance-based financing challenges?

    Ibijyanye na PBF?

    g. Others?

    Ibindi?

13. What is the contribution of MESH program in resolving these barriers?

    Ni uruhe ruhare program ya MESH yagaragaje mu gukemura izi imbogamizi?

14. In your experience, what changes have you seen since IMCI mentorship has been implemented?

Mu bunararibonye bwanyu, ni izihe mpinduka mwabonye kuva hatangira gahunda yo guhugura wigisha muri PCIME.

a. Use of IMCI protocols to assess, classify, and treat under-5 patients?

Gukoresha impapuro ngenderwaho za PCIME (protocols) mu kuvura abana bafite munsi y'imyaka itanu?

b. Health center organization (scheduling/staffing)?

Kuringaniza akazi mu Kigonderabuzima(Gahunda y'akazi n'abakozi)?

c. Availability of consultation room?

Kuboneka kw'icyumba cy'isuzumiro

d. Availability of equipments?

Kuboneka kw'ibikoresho?

e. Availability of protocols and patient charts?

Kuboneka kw' imirongo ngenderwaho(protocols) n'amafishi y'abarwayi

f. Health center management?

Imiyoborere y'ikigo nderabuzima

g. Other?

Ibindi?

<u>Acceptance and expansion Ukwemerwa no kwagura</u>

15. If you were to decide, would you recommend MESH to continue in this district?

Bibaye ngombwa mufata icyemezo, mwakwifuza ko MESH ikomeza gukorera muri aka karere?

a. Why or why not?

Niba ari yego cyangwa oya ni ukubera iyihe mpamvu?
