# Supplementary material for: Clinical mentorship to improve pediatric quality of care at the health centers in rural Rwanda: a qualitative study of perceptions and acceptability of health care workers
Source: BMC Health Serv Res. 2014 Jun 20;14:275. doi: 10.1186/1472-6963-14-275 (PMC4077561; doi:10.1186/1472-6963-14-275)
Supplement: Additional file 4 — Codebook-Nurse mentees. [file 1472-6963-14-275-S4.pdf]

Interview Guide: District Medical Director

Setting the Stage

1. In your own words, what is IMCI?

   Mbese mukoresheje amagambo yanyu, PECIME ni iki?

   a. What specific IMCI services are offered to sick children at this health center?
      Ni izihe servisi za PECIME zihabwa abana barwaye kuri iki kigo Nderabuzima?

2. How would you describe what the MESH program is?

   Ni gute mwasobanura program ya MESH icyo ari cyo?

3. What types of activities do the mentors do when they visit this health center?

   Ni iyihe mirimo abagenzuzi bahugura(mentors) bakora igihe babasuye ku kigo nderabuzima?

   a. How do IMCI mentors share their clinical knowledge during mentoring visits?

Ni gute abagenzuzi bahugura(mentors) batanga kubumenyi bafite igihe basuye ikigo nderabuzima?

   b. How do they give nurse menteesfeedback about what theyare doing well or what theycould improve?

   c. Ni gute baha abaforomo ibitekerezo kubijyanye n'ibyo bakora cyangwa ibyo bakwiriye kuvugururura

   d. How could the mentor improve their support for health centerstaff ?

   Ni gute umugenzuzi uhugura (mentor) yagombaga kuba yarafashije muburyo buruseho kuba bwiza igihe aheruka kubasura?

Communication& Skill-Building

4. How does the mentor share with you what they see is going well or what could be improved in IMC I care at this health center?

   Ni gute umugenzuzi wigisha abamenyesha ibyo abona bigenda neza cyangwa ibishobora kurushaho kunozwa muri PECIME?

   a. How does the mentor help you problem solve and come up with solutions to challenges?

   Ni gute umugenuzi wigisha(mentor) abafasha mu gukemura ibibazo no gushaka umuti kumbogamizi muhura nazo?

5. What management challenges have mentors helped resolve at this health center?

   Ni ibihe bibazo umugenzuzi wigisha(mentors)yafashije gukemura mubijyanye n'imiyoborere?

   a. Do you value the help they have been able to provide? How so?

   b. Mwaba muha ireme ubufasha batanga?sobanura?

   c. Are there ways that the mentors are <u>not</u> helpful, or could be better?

Haba hari uburyo runaka mubona abagenzuzi bahugura(mentors) badatanga ubufasha buhagije cyangwa aho bakwiriye kuvugurura?

Trust

6. How comfortable do health center staffs feel in asking mentors questions about challenges they experience?

   Mbese abakozi b'ibigo nderabuzima bumva biboroheye kubaza abagenzuzi bahugura (mentors) ibibazo bijyanye n'ingorane bahura nazo?

Mentoring vs.traditional supervision

7. How can you describe the difference between the roles of clinical mentors compared to traditional supervisors? Particularly in IMCI?

   Ni gute mwasobanura itandukaniro riri hagati y'inshingano z'abagenzuzi bahugura n'umugenzuzi basanzwe(muburyo bwari bumenyerewe kera)? Cyane mwibande kuri PCIME?

   a. In which ways is mentoring different from traditional supervision?

      Ni mubuhe buryo kugenzura uhugura (mentoring) bitandukanye n'ubugenzuzi busanzwe (muburyo bwa kera)

   b. In which ways are mentoring and traditional supervision similar?

      Ni mu buhe buryo kugenzura uhugura(encadrement) n'ubugenzuzi busanzwe byaba ari bimwe ?

Areas of improvement

8. What components of mentoring do you think need improvement?

   Ni ibihe bice bya gahunda y'igenzura uhugura(mentoring)mutekerezako byaba bikeneye kuvugururwa?

9. What changes would you suggest to improve IMCI MESH program health centers?

   Ni izihe mpinduka wifuza zarushaho kunozwa mu mikorere ya programu ya MESH muri PCIME ku bigo nderabuzima?

   a. Changes to mentoring visit structure?

      Impinduka mubijyanye n'uko isurwa rikorwa?

   b. Knowledge or skills of mentors?

      Ubuhanga n'ubumenyi bw'abagenzuzi bahugura

   c. Mentoring techniques?

      Ibijyanye na tekiniki bakoresha muguhugura no kwigiisha?

   d. Other?

      Ibindi?

Barriers to IMCI delivery & MESH contributions/ Imbogamizi muri gahunda ya MESH muri PECIME n'uruhare rwa MESH

10. What barriers do you find to IMCI delivery?

    Ni izihe mbogamizi ibigo nderabuzima bihura nazo muri gahunda ya PCIME?

    a. Nurse training and skills?

       Imbogamizi zijyanye n'ubumenyi bw'abaforomo n'amahugurwa?

    b. Systems for nurse assignments and clinic scheduling?

       Imbogamizi zijyanye n'imikorere y'ikigonderabuzima n'uburyo akazi gapangwa

    c. Paperwork and reporting challenges?

       Imbogamizi zijyanye no kuzuza impapuro n'amaraporo?

    d. Medication and equipment availability?

       Ukuboneka kw'imiti n'ibikoresho nkenerwa?

    e. MOH support?

       Ubufasha butangwa na ministeri y'ubuzima?

    f. Performance-based financing challenges?

       Ibijyanye na PBF?

    g. Others?

       Ibindi?

11. What is the contribution of MESH program in resolving these barriers?
12. *Ni uruhe ruhare program ya MESH yagaragaje mu gukemura izi imbogamizi?*

Mu bunararibonye bwanyu, ni izihe mpinduka mwabonye kuva hatangira gahunda yo guhugura wigisha muri PCIME.

a. Use of IMCI protocols to assess, classify, and treat under-5 patients?

   Gukoresha impapuro ngenderwaho za PCIME (protocols) mu kuvura abana bafite munsi y'imyaka itanu?

b. Health center organization (scheduling/staffing)?

   Kuringaniza akazi mu Kigonderabuzima(Gahunda y'akazi n'abakozi)?

c. Availability of consultation room?

   Kuboneka kw'icyumba cy'isuzumiro

d. Availability of equipments?

   Kuboneka kw'ibikoresho?

e. Availability of protocols and patient charts?

   Kuboneka kw' imirongo ngenderwaho(protocols) n'amafishi y'abarwayi

f. Health center management?

   Imiyoborere y'ikigo nderabuzima

g. Other?

Ibindi?

Acceptance and expansion/ Ukwemerwa no kwagura

13. If you were to decide, would you recommend MESH to continue in this district?

Bibaye ngombwa mufata icyemezo, mwakwifuza ko MESH ikomeza gukorera muri aka karere?

a. Why or why not?

   Niba ari yego cyangwa oya ni ukubera iyihe mpamvu?
