# Supplementary material for: Clinical mentorship to improve pediatric quality of care at the health centers in rural Rwanda: a qualitative study of perceptions and acceptability of health care workers
Source: BMC Health Serv Res. 2014 Jun 20;14:275. doi: 10.1186/1472-6963-14-275 (PMC4077561; doi:10.1186/1472-6963-14-275)
Supplement: Additional file 5 — Codebook-Others. [file 1472-6963-14-275-S5.pdf]

Interview Guide: MESH IMCI Mentors

Inyoborakiganiro: Abagenzuzi

Setting the Stage: Igice gitangira

1. What is the role of mentors in IMCI?
1. Ni akahe kamaro k'aba mentors?
   a. What are the main steps of a mentoring visit?
   Ni ibihe byiciro by'ingenzi byo kugenzura uhugura (mentoring)?
   b. How do you share your clinical knowledge during mentoring visits?

      Ni gute utanga ubumenyi mugihe uri muri gahunda y'igenzura uhugura(mentoring)?

   c. How do you give you feedback about what a nurse is doing well or what he/she could improve?

      Ni gute wungura ibitekerezo kubyo abaforomo bakoraneza cyangwa bakwiriye kunoza mu mikorere?

Communication& Skill-Building

2. Please tell me about a time when you had a difficult time communicating a new concept to a nurse.

   Mushobora kumbwira igihe byaba byakugoye kumenyesha umuforomo ijambo/inyigisho nshya.

   a. How did you realize that the nurse did not understand?

      Ni gute mwamenye ko umuforomo atumvise?

   b. What did you do or when you realized that the nurse did not understand?

      Wabigenje ute cyangwa ubigenza ute iyo ubona umuforomo atumvise?

3. What are your strategies for coaching nurses?

   Ni izihe ngamba ukoresha mu guhugura abaforomo?

   a. Do you think coaching differs from regular teaching or supervision? How so?

      Mbese utekereza ko guhugura bitandukanye no kwigisha cyangwa kugenzura bisanzwe? Sobanura?

4. How do you find your nurse mentees are in admitting when there is something they don't know, or can't remember?

   Mbese nigute umenya ko abaforomo wigisha bemera ko hari ibyo batazi cyangwa batibuka?

   a. What do you do to help the nurses feel comfortable talking about their challenges and difficulties?

      Ni iki ukora kugirango ufashe abaforomo kumva biboroheye kuvuga ibibazo n'imbogamizi bafite?

Mentoring vs.traditional supervision

5. How can you describe the difference between the roles of clinical mentors compared to traditional supervisors? Particularly in IMCI?

   Ni gute wasobanura itandukaniro hagati y'inshingano z'abagenzuzi bahugura(mentors) n'aba genzuzi muburyo busanze? Cyane muri PECIME?

   a. In which ways is mentoring different from traditional supervision?

      Ni mu buhe buryo kugenzura uhugura(mentoring) bitandukanye n'igenzura?

   b. In which ways are mentoring and traditional supervision similar?

      Ni mu buhe buryo kugenzura uhugura byaba ari kimwe n'igenzura?

Areas of improvement

6. What components of the MESH program do you think need improvement?

   Ni ikihe gice cya programu ya MESH gikeneye kunozwa?

7. What changes would you suggest to improve IMCI MESH program health centers?

   Ni iyihe mpinduka wakwifuza kugirango uvugurure gahunda ya MESH muri PECIME ku bigo nderabuzima?

   a. Changes to mentoring visit structure?

      Uko igenzura mpugura (mentoring) rikorwa?

   b. Relationships between mentors and nurse mentees?

      Imibanire hagati y'abagenzuzi bahugura (mentors) n'abaforomo?

   c. Relationships between mentors and titulaires?

      Imibanire hagati y'abagenzuzi bahugura (mentors) n'abayobozi b'ibigo nderabuzima

   d. Time spent with each nurse mentee/at each health center?

      Igihe umugenzuzi uhugura(mentor) amarana n'umuforomo kuri buri kigo nderabuzima?

   e. Other?

      Ibindi?

Barriers to IMCI delivery & MESH contributions

8. What barriers do you find to IMCI delivery?

   Ni izihe mbogamizi ubona muri gahunda yo kuvura hakoreshejwe PECIME?

   a. Nurse training and skills?

      Ubumenyi n'ubuhanga bw'umuforomo?

   b. Systems for nurse assignments and clinic scheduling?

Gahunda yo gupanga imirimo na gahunda z'abaforomo?

c. Paperwork and reporting challenges?

   Raporo n'ibindi byinshi bagomba kwandika?

d. Medication and equipment availability?

   Ukuboneka kw'imiti n'ibikoresho?

e. MOH support?

   Ubufasha bwa ministeri y'ubuzima

f. Performance-based financing challenges?

   Imbogamizi zijyanye na PBF?

g. Others?

   Ibindi?

9. What is the contribution of MESH program in resolving these barriers?

   Ni uruhe ruhare rwa programu ya MESH mu gukemura izo mbogamizi?

10. What changes have you seen since you started the IMCI mentorship at the health centers you work with?

    Mu bunararibonye ufite,ni izihe mpinduka wabonye muri gahunda y'igenzura nyigisha yatangijwe?

    b. Use of IMCI protocols to assess, classify, and treat under-5 patients?

       Gukoresha amabwiriza ngenderwaho ya PECIME?

    c. Health center organization (scheduling/staffing)?

       Gahunda y'ikigo nderabuzima(gupanga/gushyira abakozi muri servisi)

    d. Availability of consultation room?

       Ukuboneka kw'icyumba cy'isuzumiro?

    e. Availability of equipment?

       Ukuboneka kw'ibikoresho?

    f. Availability of protocols and patient charts?

    Ukuboneka kw'amabwiriza ngenderwaho n'amafishi y'abarwayi?

    g. Health center management?

       Imiyoborere y'ikiigo nderabuzima

h. Other?
   Ibindi?

Acceptance and expansion

11. If you were to decide, would you recommend MESH to continue in this district?
Ubaye uwo gufata icyemezo, wasaba ko programu ya MESH ikomeza muri aka karere?

   i. Why or why not?
      Niba ari byo cyangwa atari byo tanga ibisobanuro?
